# The Importance of Mechanical Forces for *in vitro* Endothelial Cell Biology

**DOI:** 10.3389/fphys.2020.00684

**Published:** 2020-06-18

**Authors:** Emma Gordon, Lilian Schimmel, Maike Frye

**Affiliations:** ^1^Division of Cell and Developmental Biology, Institute for Molecular Bioscience, The University of Queensland, Brisbane, QLD, Australia; ^2^Institute of Clinical Chemistry and Laboratory Medicine, University Medical Center Hamburg-Eppendorf, Hamburg, Germany

**Keywords:** blood endothelial cells, lymphatic endothelial cells, mechanotransduction, fluid shear stress, matrix stiffness, ECM - extracellular matrix, *in vitro* model culture system, (lymph-)angiogenesis

## Abstract

Blood and lymphatic vessels are lined by endothelial cells which constantly interact with their luminal and abluminal extracellular environments. These interactions confer physical forces on the endothelium, such as shear stress, stretch and stiffness, to mediate biological responses. These physical forces are often altered during disease, driving abnormal endothelial cell behavior and pathology. Therefore, it is critical that we understand the mechanisms by which endothelial cells respond to physical forces. Traditionally, endothelial cells in culture are grown in the absence of flow on stiff substrates such as plastic or glass. These cells are not subjected to the physical forces that endothelial cells endure *in vivo*, thus the results of these experiments often do not mimic those observed in the body. The field of vascular biology now realize that an intricate analysis of endothelial signaling mechanisms requires complex *in vitro* systems to mimic *in vivo* conditions. Here, we will review what is known about the mechanical forces that guide endothelial cell behavior and then discuss the advancements in endothelial cell culture models designed to better mimic the *in vivo* vascular microenvironment. A wider application of these technologies will provide more biologically relevant information from cultured cells which will be reproducible to conditions found in the body.

## Introduction

Blood and lymphatic vessels are critical components of the vascular system, controlling the transport, delivery and recycling of nutrients and waste to all tissues in the body. The blood vascular system is comprised of a closed circulatory network of arteries, veins and capillaries. Arteries transport oxygenated blood with gases, nutrients, metabolites and immune cells to the organs, while veins return oxygen-poor blood to the heart. In contrast to the blood vascular system, lymphatic vessels are comprised of a blind-end, unidirectional vascular network of lymphatic collecting vessels and capillaries. Due to their specialized button-like cell junctions, lymphatic capillaries are able to take up fluid, macromolecules and immune cells. The lymph is then transported through collecting vessels that are equipped with zipper-like junctions and drained back into the venous circulation (Potente and Makinen, 2017).

As a result of their unique functions, each vessel sub-type is subjected to unique mechanical stresses. They are comprised of specialized subtypes of endothelial cells (ECs) with unique properties and genetic profiles, allowing them to perform their specific function (Potente and Makinen, 2017). Not only does each vessel have unique ECs, the EC properties also differ across tissue beds. For example, blood vascular ECs are continuously aligned in most tissues, but fenestrated in tissues involved in filtration and secretion (kidney and intestinal mucosa) or discontinuous in sinusoidal vascular beds (liver and bone marrow) [reviewed in detail by Augustin and Koh (2017)]. Lymphatic endothelial cells also display heterogeneity across tissue beds, with specialized Schlemm’s canal vessels found in the eye and meningeal lymphatics found in the brain [reviewed in detail by Petrova and Koh (2018)].

In addition to the heterogeneity of ECs, vessels are surrounded by a wide range of support structures with differing mechanical properties. They may be surrounded by supportive mural cells [such as pericytes and smooth muscle cells (SMCs)] and varying components of extracellular matrix, which is comprised of basement membrane (BM) and the interstitial matrix (occupying/filling the interstitial space). Large arteries and veins are characterized by a continuous lining of BM and layers of mural cells, whereas lymphatic collecting vessels only exhibit a thin BM layer and sparse SMC support. Lymphatic capillaries lack mural cell support and are characterized by a discontinuous or absent BM (Potente and Makinen, 2017). These features allow each vessel subtype to maintain its integrity while performing its unique function.

Much of the pioneering work characterizing EC structure and function was performed using cells grown *in vitro*. It is beginning to be appreciated that ECs within their *in vivo* environment differ greatly to those that are cultured in static two-dimensional or three-dimensional (2D/3D) settings. Indeed, the physical forces that ECs are subjected to *in vivo*, such as fluid shear stress, stiffness and stretch, greatly influence how these cells function. Here, we will briefly summarize what is known about the mechanical forces that regulate EC function and further discuss existing and emerging techniques to model these forces *in vitro*. By growing cells in mechanically relevant conditions, we can more accurately predict cellular behavior in physiological environments.

## Mechanotransduction of Fluid Shear Stress

Blood ECs lining the vessel wall are subjected to mechanical forces due to the friction between the ECs and the blood being pumped from the heart, known as fluid shear stress (FSS). The effects of FSS depends on a multitude of factors, such as the magnitude and direction of flow and the speed and strength of pump pulses (Baeyens et al., 2016). Shear stress can be steady laminar (where fluid moves in one direction at a steady magnitude), disturbed laminar (where flow is separated, recirculates and subsequently reattaches) oscillatory (where laminar flow fluid moves in a bidirectional manner) and turbulent (where flow is chaotic and moves in all directions) (Chatzizisis et al., 2007). All FSS may be pulsatile, where magnitude of flow is varied. Both oscillatory and turbulent flow may also be classified as disturbed. These different stresses play a key role in regulating vascular physiology, as alterations to FSS parameters or changes in the EC response can lead to a range of vascular pathologies [reviewed in Baeyens et al. (2016)]. Indeed, disturbed flow is known to have a wide range of effects on ECs, inducing changes in gene expression, cell shape and inflammatory profiles. This can lead to diseases such as atherosclerosis and pulmonary arterial hypertension. For a more detailed view on the effects of disturbed flow in the blood vasculature, please see the recent review by Souilhol et al. (2020).

As arteries transport blood away from the heart, they are subjected to high FSS (10–50 dyn/cm^2^) which is highly pulsatile, whereas veins are subjected to a more consistent flow with forces around 10-fold less (Paszkowiak and Dardik, 2003). Unlike blood vessels which are controlled by a central pump, lymphatic capillaries are exposed to interstitial fluid flow, thus are exposed to the lowest FSS, approximately 10-fold less than blood vessels (Dixon et al., 2006). The uptake of interstitial fluid and resulting forces are guided by hydrostatic and osmotic pressure between lymphatic vessels, blood vessels and the interstitial space [known as Starling forces (Swartz and Fleury, 2007; Levick and Michel, 2010)]. Collecting lymphatic vessels have an intrinsic pumping capacity and smooth muscle cell coverage, thus are exposed to greater FSS than capillaries to ensure efficient transport of the lymph back to the venous circulation (Scallan et al., 2016). Here, we will discuss how ECs sense and respond to these different forces to generate and maintain a functional vascular network.

### Flow Mediated Mechanosensory Hubs at Junctions

In order for ECs to respond to FSS, they require mechanisms to sense changes in flow conditions. This may be done via cilia, glycocalyx, ion channels, G-proteins or protein kinases (Hahn and Schwartz, 2009; Goetz et al., 2014; Wang et al., 2015; Givens and Tzima, 2016; Chistiakov et al., 2017). Perhaps the most well characterized mechanism of FSS sensing is through cell surface receptor ‘mechanosensory hubs’ found at cell–cell junctions [otherwise known as adherens junctions (AJs)]. Receptors within these AJ hubs include PECAM-1 (CD31), Vascular endothelial (VE)-cadherin, VEGFR2 and VEGFR3 (Tzima et al., 2005; Conway et al., 2013; Coon et al., 2015). Most central to this hub is PECAM-1, which is a direct sensor of shear stress tension (Tzima et al., 2005). Increased tension on PECAM-1 via steady laminar FSS [12 dyn/cm^2^ for 16 hours (h)] triggers association of this cell surface receptor with the vimentin cytoskeleton, which permits transmission of force from myosin to PECAM-1 (Conway et al., 2013). These tensile forces on PECAM-1 trigger the activation of Src family kinases (SFK), which in turn phosphorylate VEGF receptors in a ligand independent manner. Phosphorylated VEGFRs then activate downstream signaling cascades, including the PI(3)K and NFκB pathways (Tzima et al., 2005; Coon et al., 2015). Within this complex, VE-cadherin acts to assemble VEGFRs at the junction through its transmembrane domain, facilitating their activation by SFK (Coon et al., 2015).

While known to be a critical component of AJ mechanosensory hubs and a key controller of vascular integrity and sprouting (Coon et al., 2015; Schimmel and Gordon, 2018), the precise role of VE-cadherin as a sensor of tension in response to shear stress remains undefined. In contrast to PECAM-1, when steady laminar FSS is applied to VE-cadherin (15 dyn/cm^2^), tension is reduced within 2 min (Conway et al., 2013). Interestingly, 2 min at 15 dyn/cm^2^ is sufficient to induce tension on PECAM-1 (Conway et al., 2013). *In vivo*, VE-cadherin tension in vessels under high levels of FSS (arteries) is lower than that observed in low shear, immature vessels (Lagendijk et al., 2017). However, VE-cadherin can be phosphorylated in response to PECAM-1-SFK on its Tyr658 site (Conway et al., 2017). This results in dissociation of p120-catenin and association with polarity protein LGN, which subsequently activates inflammatory pathways at sites of disturbed flow. Additional research is required to fully elucidate the mechanosensory role of VE-cadherin in response to FSS in physiological settings.

### Flow Mediated Mechanosensory Hubs at Cell-Matrix Interfaces

In addition to junctions, mechanosensory hubs are found at sites of cell-matrix adhesion [otherwise known as focal adhesions (FAs)]. FAs are comprised of extracellular matrix (ECM) binding proteins known as integrins, which are coupled to the cytoskeleton through actin binding proteins, including the mechanosensors vinculin and talin (Bays and DeMali, 2017; Goult et al., 2018). FAs are vital components of the EC mechanosensory complex, mediating a wide range of signaling cascades in response to traction forces and FSS (Katsumi et al., 2004; Yurdagul and Orr, 2016). However, while FAs are remodeled in response to FSS and ECs display larger FA under steady laminar flow (17 dyn/cm^2^) than those under disturbed flow after 14 h (Ting et al., 2012), whether FA proteins themselves can physically sense changes in tension in response to FSS remains unclear.

Within FAs, integrins play a central role in transducing chemical and physical signals from their surroundings by interacting and binding to their adhesion ligands presented by the BM. For a detailed overview of integrins and their ECM interactions, please see the following reviews (Ross et al., 2013; Hamidi and Ivaska, 2018). Notably, integrin can mediate bi-directional signaling. “Inside-out” signaling activates the ligand binding function of integrins (for example when FSS is sensed and subsequently transduced inside the EC) and “outside-in” signaling mediates cellular responses (for example in response to changes in ECM stiffness) leading to changes in cell spreading, retraction, migration, and proliferation (Hamidi and Ivaska, 2018). Although integrins do physically respond to FSS, albeit at a significantly lower degree compared to their response to traction forces (Katsumi et al., 2004), whether they confer biologically relevant tension changes in response to FSS remains unclear. However, they are activated downstream of PECAM-1 and PI(3)K activity (Collins et al., 2012; Russell-Puleri et al., 2017), revealing they may indeed involved in a FSS mechanosensory complex. The Integrin-actin linker protein talin is a well-established traction force mechanosensor (Kumar et al., 2016, 2018; Goult et al., 2018). Yet similar to integrins, whether it confers physical tension sensing capacity in response to FSS remains unclear. Talin activates integrins by binding to the β-tail (Yurdagul and Orr, 2016) and loss of talin results in impaired integrin activation (Monkley et al., 2000). EC-specific knockout of either integrin-β1 itself or talin (leading to loss of integrin-β1 activation) leads to perturbed VE-cadherin localization and loss of junctional integrity (Yamamoto et al., 2015; Pulous et al., 2019). This reveals that FAs are intimately associated with AJs, potentially through their attachment to the cytoskeleton. However, it may also suggest that integrins and talin may not be FSS tension sensors, but are simply activated downstream of AJ mechanosensory hubs though the physical link with the cytoskeleton or by downstream signaling pathways. More work is required to elucidate the FSS tension sensing capacity of integrins and their associated actin-binding proteins.

Recently, it was reported that guidance receptor PlexinD1 is a mechanosensor, and upon loss of PlexinD1 cells fail to align after 24 h steady laminar FSS (12 dyn/cm^2^). This occurs through a PlexinD1-Neuropilin-1 complex with VEGFR2, which acts as a mechanosensor upstream of the PECAM-1 mediated junctional complex and integrin-based focal adhesions (Mehta et al., 2020). If PlexinD1 is held in its ring-like conformation it cannot act as a mechanosensor, suggesting it is not only the expression, but also the structure, of each mechanosensory component which confers its role.

### Sensing Flow Dynamics During Vessel Sprouting and Remodeling

As blood and lymphatic vessels sprout, mature and remodel, they become subjected to different FSS. Therefore, EC mechanosensors need to be able to adapt to each vessel’s physiological requirements. During formation of the initial blood vascular plexus *in vivo*, angiogenic sprouting is more likely to occur at sites of low FSS (Ghaffari et al., 2015) and vascular fusion is induced by steady laminar low FSS and VE-cadherin phosphorylation (induced at 1.8 dyn/cm^2^ but not 10 dyn/cm^2^ for 4 h) (Caolo et al., 2018). Later in development during vascular remodeling, in vessels with low FSS (such as capillaries) ECs are less likely to align against the direction of flow than ECs in regions of high FSS (such as arteries) (Franco et al., 2015). This prevents polarity-induced migration into regions of high flow and subsequent vessel regression. ECs deficient in canonical Wnt signaling display increased sensitivity to FSS-induced regression *in vivo* or after being subjected to steady laminar FSS in culture (20 dyn/cm^2^ for 4 h) (Franco et al., 2016), suggesting that a FSS ‘setpoint’ controlling EC polarity and vascular stability is modulated by Wnt. These ‘setpoints’ define the optimal FSS exposure for normal vascular function, whereby if FSS is above or below the setpoint, vascular abnormalities occur. Interestingly, loss of Wnt signaling leads to reduced sprouting capacity (Korn et al., 2014; Carvalho et al., 2019), yet whether this is guided through altered sensitivity to a low FSS setpoint remains unclear.

In addition to FSS setpoints being defined during EC polarity and remodeling, they must be specified across different vessel sub-types in order for each vessel to exert its biological function. As blood vessels are exposed to higher FSS in the body than lymphatic vessels, blood ECs become misaligned and activate NFκB at much higher steady laminar FSS levels (25 dyn/cm^2^ and over for 16 h) than that of lymphatic ECs (10 dyn/cm^2^ and over for 16 h) (Baeyens et al., 2015). This allows blood vessels to be exposed to higher rates of FSS without causing inflammation and disease. This is a reflection of vessel physiology – lymphatics are exposed to significantly lower FSS *in vivo* as their function is to transport interstitial fluid back to venous circulation. The FSS setpoint in blood EC versus lymphatic EC is mediated though expression of VEGFR3, which is enriched in lymphatic ECs compared to blood ECs (Kaipainen et al., 1995). The elevated expression of VEGFR3 in lymphatic EC causes them to respond to FSS at lower levels to that of blood ECs, and reducing VEGFR3 expression in lymphatic ECs increases the FSS setpoint (Baeyens et al., 2015). Conversely, increasing VEGFR3 expression in blood EC decreases the FSS setpoint, causing induction of inflammatory profiles at FSS levels seen in lymphatic EC.

Although FSS is lower in lymphatic compared to blood vessels, there are still physiologically relevant fluctuations in flow that drive genetic and morphological changes in lymphatics. During early development, functional lymphatic drainage can be detected as early as embryonic day (E) 11.5 (Planas-Paz et al., 2012) suggesting that induction of FSS might contribute to the development of the primary lymphatic plexus by inducing lymphatic EC proliferation. Indeed, steady laminar FSS has been shown to induce proliferation via ORAI1 and KLF2/4-mediated upregulation of VEGF-A, VEGF-C, FGFR3 and p57 (2 dyn/cm^2^ for 12–24 h) (Choi et al., 2017a) and downregulation of NOTCH1 activity (2 dyn/cm^2^ for 12–24 h) (Choi et al., 2017b). Lymphatic valve development is predominantly initiated in areas of disturbed flow, and oscillatory flow (4 dyn/cm^2^ for 48 h) has been shown to reproduce a lymphatic valve EC phenotype *in vitro* (Sabine et al., 2012). Oscillatory flow but not steady laminar flow (4 dyn/cm^2^ for 48 h) induces expression of the transcription factor GATA2 in valve-forming lymphatic ECs, which in turn upregulates expression of the lymphatic endothelial transcription factors PROX1 and FOXC2 (Kazenwadel et al., 2015), both of which are key regulators of valve formation. In agreement, loss of lymphatic flow in a mouse model of CLEC2-deficiency (in which blood backfills the lymphatic network and thereby blocks lymph flow) results in a phenotype identical to that observed upon loss of FOXC2 (Petrova et al., 2004; Sweet et al., 2015). In the postnatal lymphatic vasculature, FOXC2 is necessary to maintain collecting vessel quiescence and stability in valve areas by linking oscillatory flow responses to cell junction stabilization and cell-cycle arrest (Sabine et al., 2015), revealing flow dynamics as key regulators of the formation and maintenance of a functional lymphatic system.

### Changes in Membrane Tension by FSS

Interestingly, FSS can induce changes in membrane curvature, leading to activation of a range of cell surface receptors including calcium and ion channels. The ion channel Piezo1, which is sensitive to changes in membrane tension (Lewis and Grandl, 2015; Cox et al., 2016), is also required for FSS sensing (Ranade et al., 2014; Albarran-Juarez et al., 2018), thereby coupling FSS and membrane stretch. Piezo1 regulates vascular permeability in the lungs by inducing VE-cadherin internalization, and subsequent leakage, in response to increased hydrostatic pressure (Friedrich et al., 2019). Piezo1 is also mechanically activated in lymphatic vessels and is required for lymphatic valve formation (Nonomura et al., 2018) by sensing disturbed flow to induce GATA2 and FOXC2 expression (Choi et al., 2019). Although not as widely studied, ion channels such as TRPV4 have also been implicated in FSS sensing to regulate vascular permeability (Thorneloe et al., 2012). Not only is membrane curvature altered, but membrane fluidity changes upon the onset of shear stress, which may directly induce the difference in tension that opens up channels such as Piezo1. However, these changes occur in biologically inactive lipid membranes, thus may be a basic physical phenomena that can transduce shear stress (Yamamoto and Ando, 2013).

In addition to Piezo1, caveolae (invaginations on the cell membrane) are sensitive to changes in membrane tension and are known to be altered in response to FSS (Shihata et al., 2016). ECs flatten their caveolae in response to increased osmotic pressure to allow for membrane ‘stretching,’ which may be a survival mechanism by increasing cell surface area (Sinha et al., 2011; Parton and del Pozo, 2013). Interestingly, a recent study found caveolae are abundant in arterial ECs of the central nervous system and are responsible for transmitting signals from the neurovascular unit to adjacent SMC to induce vasodilation (Chow et al., 2020). This was proposed to be through the ability of caveolae to cluster TRPV4 ion channels (Goedicke-Fritz et al., 2015) rather than mediating changes in arterial membrane stretch. Precisely how physical stretch by FSS alters membrane components in ECs remains largely unknown.

## Mechanotransduction of Extracellular Matrix (ECM) Stiffness

Extracellular matrix stiffness has been shown to guide the behavior of ECs to form the initial functional vascular network. Embryonic stem cells (ESCs) depend on the mechanical environment of the stem cell niche, as the fate of vascular progenitor cells (VPC) was shown to be dependent on substrate stiffness, with EC lineages favoring softer substrates (10 kPa) and SMC lineages favoring stiffer substrates (plastic, GPa range) (Wong et al., 2019). During early lymphatic development, venous lymphatic EC progenitors laminate from the cardinal vein and encounter very soft surrounding tissue (0.27 kPa) while they migrate dorsally to form primary lymphatic structures (Frye et al., 2018). This value matches those measured in early chicken embryos (0.3 kPa) (Majkut et al., 2013) and adult brain (0.33 kPa) (Georges et al., 2006) but are significantly lower compared to values from most other adult tissues, such as muscle (around 12 kPa) (Engler et al., 2004) or bone (GPa range) (Rho et al., 1993). Upon remodeling and maturation, ECs form the inner layer of blood and lymphatic vessels and are attached to the underlying 2D ECM environment. The composition and mechanical properties of the ECM differ along vascular trees, across tissues and during development and disease states (Di Russo et al., 2017).

The range of venous tissue stiffness in mammals is about 3–50 kPa (Xue et al., 2017; Frye et al., 2018), which is positioned between the stiffness of the epithelium and cartilage. Unlike veins, arteries must be able to withstand higher blood pressure and are surrounded by several layers of smooth muscle cells and connective tissue (Corada et al., 2014). Therefore, arteries are stiffer, ranging from about 50–150 kPa (Kohn et al., 2015). Differences in ECM stiffness experienced by endothelial progenitor cells (EPCs) have been implicated in the regulation of arterial-venous differentiation *in vitro* (Xue et al., 2017). In contrast to EPCs cultured on venous substrate stiffness (7 kPa), EPCs cultured on arterial substrate stiffness (128 kPa) showed an increase in expression of the arterial marker EphrinB2 (Xue et al., 2017).

The stiffness values experienced by the endothelium must be interpreted with caution as substantially different measurement techniques have been employed to generate values [reviewed by Kohn et al. (2015) and Guimarães et al. (2020)]. Macroscale techniques, such as Shear Wave Elastography (SWE) (Maksuti et al., 2016) generally analyze the entire vessel structure as a uniform material, and do not take into account cell-scale differences of the individual vessel components, such as the endothelial or SMC layer or the surrounding ECM. Furthermore, macroscale techniques typically provide values of the stiffest component of a vessel structure. Therefore, these data underscore the importance of analyzing mechanical properties of individual vessel components using microscale techniques, such as Atomic Force Microscopy (AFM) (Alsteens et al., 2017; Frye et al., 2018) and Scanning Ion Conductance Microscopy (SICM) (Schaffer, 2013). It is important to attempt comprehensive measurements of *in vivo* ECM stiffness experienced by ECs to accurately choose the stiffness values for studies on EC-type or organ-specific vasculature.

### EC-Induced ECM Deformation

Adherent cells sense ECM stiffness by probing their extracellular environment, resulting in substrate displacements when substrates are compliant. The measurement of compliant ECM substrate deformations (and calculation of deformation-based metrics) via Traction Force Microscopy (TFM) (Califano and Reinhart-King, 2009) or 4D displacement microscopy (Vaeyens et al., 2020) can provide quantitative information on cell-ECM mechanical interactions. For example, by employing 4D displacement microscopy, it has been demonstrated that ECM substrate deformations are spatio-temporally correlated with sprout-morphological dynamics during sprouting angiogenesis (Vaeyens et al., 2020). Interestingly, protrusions from extending sprouts increase their pulling forces, while retracting protrusions reduce pulling.

*In vitro* ECM substrate deformation analysis can provide insight into how ECs exert forces into their surroundings. However, which ECM components or EC-surrounding cells are responsible to generate the absolute *in vivo* ECM stiffness that counteracts those deformation forces is poorly defined. The BM underlying ECs in most mature vessel types is a very thin layer (30–500 nm) and is comprised of ECM matrix molecules such as elastin, collagen, enactin/nidogen, heparan-sulfate proteoglycans, and laminin (Liliensiek et al., 2009). Interestingly, a study by Sen et al. (2009) showed that cells can “feel” up to several micrometers deep into a compliant substrate. Consistently, induction of EC network formation on compliant substrate (0.4 kPa) was prevented on very thin compliant substrates (<20 μm) as ECs sensed the stiffness of the underlying coverslip (Davidson et al., 2019). These findings suggest that absolute *in vivo* ECM stiffness experienced by ECs might rather be generated by several different EC-surrounding structures, including the BM, the interstitial matrix and, importantly, the adjacent cells.

### Mechanosensing of ECM Stiffness

Similar to their FSS mechanotransduction capacity, FAs are fundamental mechanotransducers of ECM-endothelial stiffness. FAs propagate ECM stiffness stimuli to the actin cytoskeleton, resulting in cytoskeletal rearrangements that can lead to an immediate adaptation of cells to their physiological environment, such as alignment (Maniotis et al., 1997) or translocalization of proteins to the nucleus in order to modify the genetic program and thereby cellular behavior [reviewed in detail in Martino et al. (2018)]. Within FAs, the expression of integrins is highly cell type- and substrate-specific, and this integrin diversity can regulate intracellular signaling cascades in response to different mechanical stimuli (Seetharaman and Etienne-Manneville, 2018). In epithelial cells, an increase in matrix stiffness (0.2 kPa vs. GPa range) regulates the expression of β1 integrin and Cav1 to regulate FA assembly and turnover (Yeh et al., 2017). Similarly, ECs matrix stiffening (3 kPa vs. 70 kPa) has been shown to induce β1 integrin activity (Bastounis et al., 2019). Strong changes in expression of β1 integrin were not observed in this study, however, analysis of ECs on softer matrices were not included.

Intracellularly, FAs are dynamically associated with a variety of proteins that can sense and/or transduce force, such as focal adhesion kinase (FAK), vinculin and talin (Martino et al., 2018). FAK is one of the first proteins recruited to FAs in response to extracellular mechanical stimuli. In fibroblasts, autophosphorylation of FAK is required for induction of adhesion and integrin-binding responses (Michael et al., 2009). Furthermore, FAK activity is necessary to regulate directed migration of fibroblasts toward stiffer substrates (Plotnikov et al., 2012) and to induce matrix stiffness-mediated translocation of the mechanotransducer Yes-associated protein (YAP) to the nucleus of hepatic stellate cells (Lachowski et al., 2018). The mechanosensor talin is a 270 kDa protein composed of an N-terminal globular head, a flexible rod domain and C-terminal helices. It binds to the cytoplasmic domain of integrin β-subunits and regulates integrin signaling in a switch-like behavior via the helix bundles of its flexible rod domains (Goult et al., 2018). It has been suggested that individual domains open at different tension levels, exerting positive or negative effects on different protein interactions (Goult et al., 2018). For example, force loading of talin leads to the exposure of cryptic hydrophobic binding sites, which can subsequently bind vinculin (Rahikainen et al., 2017). As increasing force is applied to talin, more bundles are unfolded, revealing additional vinculin binding sites and thus activation of an increasing number of vinculin molecules (Haining et al., 2016). These FA molecules have been extensively studied as general tension sensors/transducers in non-endothelial cell types (Provenzano et al., 2009; Zhou et al., 2017; Martino et al., 2018). It will be important to study the expression and function of those proteins specifically in the context of matrix stiffness-regulated EC behavior.

After they have been engaged downstream of integrins, FAs propagate mechanical stimuli to the actin cytoskeleton. Culturing ECs on different stiffness matrices leads to a substantial remodeling of the actin cytoskeleton (Jannatbabaei et al., 2019). With increasing matrix stiffness (3 kPa, 12 kPa, and 1.5 MPa), actin cytoskeleton remodeling becomes more organized, with an increasing amount of actin stress fibers. Interestingly, increasing matrix stiffness translates to increased EC stiffness in 2D (1.7 kPa vs. 9 kPa) and 3D (0.125 kPa vs. 0.5 kPa) environments (Byfield et al., 2009; Jannatbabaei et al., 2019). The actin cytoskeleton is connected to VE-cadherin via its intracellularly associated proteins β-catenin and α-catenin (Lampugnani et al., 1995). Tension-induced actin remodeling tightly controls assembly and disassembly of VE-cadherin-based junctions (Oldenburg and de Rooij, 2014). Cytoskeletal pulling at the VE-cadherin complex also recruits the FA tension sensor protein vinculin via α-catenin to reinforce endothelial junctions (Huveneers et al., 2012; Daneshjou et al., 2015).

Besides a direct effect on endothelial junction stability, cytoskeletal mechanotransduction can result in structural modification of membrane-bound or cytoplasmic proteins and their subsequent shuttling to the nucleus. β-catenin is not only a structural protein within the VE-cadherin adhesion complex, but can also shuttle to the nucleus upon cytoskeletal remodeling to act as a transcriptional co-activator. In chondrocytes, β-catenin expression is induced and localized to the nucleus in a β1-integrin/FAK dependent manner in cells seeded on stiff matrices (100 kPa vs. 0.5–1 kPa) (Du et al., 2016). Furthermore, β-catenin was shown to localize to the nucleus in valvular ECs grown on stiff matrices (50 kPa vs. 5 kPa) (Zhong et al., 2018). Another class of nuclear shuttling proteins consists of YAP and WW Domain-Containing Transcription Regulator Protein 1 (WWTR1/TAZ), which are downstream effectors of the Hippo pathway (Zhong et al., 2018). YAP/TAZ are shuttled to the nucleus in lymphatic ECs grown on stiff substrates (25 kPa vs. 0.2 kPa) as evident by the induction of their target genes CTGF and ANKRD1 (Frye et al., 2018). YAP/TAZ function has been extensively studied in the development of the vasculature (Kim et al., 2017; Sakabe et al., 2017; Wang et al., 2017b; Neto et al., 2018; Cho et al., 2019; Sivaraj et al., 2020). Precisely how changes in ECM stiffness experienced by ECs might regulate those processes remains to be elucidated.

### ECM Stiffness and Endothelial Proliferation and Migration

During angiogenesis, VEGFR2 activation and internalization induces proliferation of ECs to enable nascent blood vessels and tumor vessels to expand (Simons et al., 2016). In blood and lymphatic ECs, proliferation is decreased on soft 2D substrates and enhanced on stiff 2D substrates. Sub-confluent HUVECs grown on stiffer substrates (10 kPa vs. 1 kPa) increase VEGFR2 internalization, mediated via Rho activity and actin contractility (LaValley et al., 2017). Additionally, expression of Septin9, a negative upstream effector of RhoA, is increased in ECs grown on soft 2D substrates (1.72 kPa vs. 21.5 kPa), attenuates Src/Vav2 phosphorylation and inhibits RhoA-dependent EC proliferation (Yeh et al., 2012). However, in a confluent endothelial monolayer, stiffness-enhanced VEGF signaling is no longer observed (LaValley et al., 2017), suggesting this mechanism is unique to actively proliferating cells and angiogenic processes.

In contrast, maximal VEGFR2 expression and capillary blood vessel formation in an *in vivo* 3D Matrigel implant assay was detected in a relatively soft microenvironment of 0.8 kPa (Mammoto et al., 2009). Similarly, lymphatic ECs leave the stiffer cardinal vein (4 kPa) to migrate through a very soft 3D environment (0.27 kPa) (Frye et al., 2018). The decrease in matrix stiffness experienced by those cells induces an increase in VEGFR3 expression that is necessary to allow efficient migration. As has been suggested by Mammoto et al. (2009), a difference in migratory and sprouting response to matrix stiffness could be explained by differential EC requirements on a 2D ECM versus a 3D ECM environment. This hypothesis is further supported by the finding that sprouting of blood ECs is increased in synthetic 3D hydrogels with lower matrix crosslinking, which is accompanied by a decrease in matrix stiffness (1 kPa vs. 6 kPa) (Trappmann et al., 2017).

Furthermore, it is important to note that *in vitro* studies have been performed using different definitions of a stiff substrate. Comparing the absolute kPa values reveals that ‘stiff’ ECM values (such as 4 kPa) are used as the ‘soft’ ECM standards in other studies (Canver et al., 2016). This underscores the necessity to comprehensively measure *in vivo* stiffness of blood and lymphatic vasculature to better define the ranges that can be adapted for *in vitro* studies.

### ECM Stiffness and Disease

More than 80% of all cardiovascular deaths in developed countries occurs in the population aged 65 or older, making aging a major risk factor for cardiovascular disease. It is well established that age-induced stiffening of the ECM in the intima alters the function of blood vessels. These alterations of ECM stiffness affect EC health, resulting in inflammation, hypertension and ultimately disease progression in disorders such as atherosclerosis and pulmonary arterial hypertension (PAH) (Zieman et al., 2005; Ley et al., 2007; Wang and Chesler, 2011; Marti et al., 2012; Tan et al., 2014). Indeed, increased vascular ECM stiffness is induced upon aging and/or inflammation, as revealed by rigidity measurements using murine aortas of different ages (Huynh et al., 2011) and murine and human atherosclerotic plaques (Tracqui et al., 2011; Chai et al., 2013). Vascular calcification is often observed in human atherosclerotic plaques and is known to induce EC dysfunction, which is one of the main characteristics of arterial aging (Lee and Oh, 2010). This calcification seems to be irreversible, with a number of studies attempting to inhibit or reverse ECM stiffening being unsuccessful (Little et al., 2005; Hope and Hughes, 2007; Zieman et al., 2007; Willemsen et al., 2010). Hence, current research has shifted toward investigating the translational effects of ECM stiffness on ECs, to explore the possibilities of reversing the EC response to increased ECM stiffness, instead of directly targeting ECM stiffening.

In the context of cancer biology, it is well appreciated that a solid tumor environment is stiffer than that of healthy tissue (Levental et al., 2009). How tumors overcome the mechanical barrier of a stiffer environment to promote tumor angiogenesis is likely through matrix metalloproteinase (MMP) signaling, which was found to be induced in ECs cultured in stiffer 3D collagen gels (Levental et al., 2009). Endothelial dysregulation results in vascular leakage due to a loss of cell junction integrity. In tumors, this dysregulation has been largely attributed to an increase in pro-angiogenic growth factors. However, tumor ECM stiffness has been shown to also play a central role in promoting a characteristically leakier tumor vasculature (Bordeleau et al., 2017). Sub-confluent ECs on compliant substrates assemble spontaneously into networks reminiscent of an angiogenic process (Califano and Reinhart-King, 2008). Therefore, sub-confluent ECs grown on soft substrates (0.2 kPa) form continuous VE-cadherin-positive junctions, which are highly discontinuous and punctuated compared to those grown on stiff, tumorigenic substrates (10 kPa) (Bordeleau et al., 2017).

Mechanistically, increased matrix stiffness activates FAK, resulting in Src localization to cell–cell junctions and subsequent Src-mediated VE-cadherin phosphorylation (Wang et al., 2019). Interestingly, loss of VE-cadherin function activates intercellular force transduction signals that increase integrin-dependent cell contractility, disrupting both cell–cell and cell-matrix adhesions. Increased substrate stiffness (40 kPa vs. 1.1 kPa) further exacerbates these effects (Andresen Eguiluz et al., 2017). Tumor matrix stiffening not only impacts angiogenesis and tumor vessel leakage, but is also implicated in regulating metastasis. Matrix stiffening from 0.4 kPa to 22 kPa induced matricellular protein CCN1, which in turn upregulates N-cadherin levels on the EC surface to facilitate cancer cell binding to the endothelium and intravasation into the vessel lumen (Reid et al., 2017). These data suggest that identifying and targeting EC behavior in response to altered stiffness in solid tumors could provide novel therapeutic approaches to normalize tumor vasculature.

### Endothelial Cellular Stiffness and Leukocyte *Trans*-Endothelial Migration (TEM)

It has become clear that changes in ECM stiffness can directly regulate EC internal stiffness and/or expression of endothelial adhesion molecules, which is correlated with altered leukocyte extravasation (Stroka and Aranda-Espinoza, 2010, 2011; Schimmel et al., 2018; Chen et al., 2019). Whereas differences in ECM stiffness directs EC migration during angiogenesis, in the case of leukocyte *trans*-endothelial migration (TEM), it is the internal EC stiffness that guides the direction of leukocyte migration. The internal stiffness of ECs is regulated by crosslinking of ICAM-1 to the actin cytoskeleton via actin binding proteins (ABPs) α-actinin and cortactin (Schaefer et al., 2014). DLC-1, a Rho GTPase Activating Protein induced by ECM stiffness, is able to stabilize this crosslinking (Schimmel et al., 2018). Loss of DLC-1 in ECs cultured on stiff substrates results in leukocyte transmigration kinetics similar to those observed for ECs cultured on soft substrates, revealing DLC-1 as a critical transmitter of ECM-induced EC stiffness required for TEM. These changes in TEM and EC internal stiffness caused by the ECM are observed in inflammatory diseases, as in areas of increased ECM stiffening (such as atherosclerotic lesions or in the lung microvasculature of PAH patients) increased leukocyte extravasation is observed (Dorfmuller et al., 2003).

Besides the effects of ECM stiffness on leukocyte TEM via changes in EC internal stiffness, it has been shown that ECM stiffness regulates leukocyte and monocyte transmigration via microRNAs. Expression of adhesion molecules ICAM-1 and VCAM-1 on ECs, both essential for leukocyte and monocyte adhesion, is regulated by miR-222 and miR-126, respectively (Harris et al., 2008; Ueda et al., 2009). ECs cultured on high stiffness substrates (20 kPa) showed reduced migration of monocytes, which correlated with higher levels of miR-222 and miR-126 to inhibit endothelial ICAM-1 and VCAM-1 expression compared to soft (8 kPa) and diseased (40 kPa) stiffness values (Chen et al., 2019). This suggest that matrix stiffness exerts a biphasic regulation of endothelial adhesion molecules ICAM-1 and VCAM-1, which promotes EC-monocyte interactions both in healthy (soft) and diseased (stiff) environments.

## Mechanotransduction of Endothelial Cell Stretch

While much focus has been placed on the role of FSS sensing and cell-ECM interactions, the role of cyclic stretch (CS) on endothelial cell function is far less studied. This is despite its intricate link to matrix stiffness, as a stiffer matrix will in principle result in a reduction of cyclic stretch. ECs are constantly exposed to cyclical stretch due to dilation across the vessel wall as a result of blood pumping, respiration in the lungs or uptake of interstitial fluid in lymphatics. This stretch of the EC membrane results in activation of signaling pathways and physiological responses, such as sprouting, permeability and remodeling (Jufri et al., 2015).

### Stretch in Blood Endothelial Cells

To date, much of our knowledge on how vascular ECs respond to stretch comes from cultured cells. In blood ECs, normal stretch is 5% elongation and pathological stress is 18% elongation, with acute stretch being 15 min and stretch being chronic 48 h (Birukov et al., 2003). Stretch has been associated with a range of pathways, many of which result in loss of barrier integrity. Even after short term exposure to CS, ECs become significantly more prone to leakage in response to thrombin or VEGF, linked to an increase in Rho GTPase activity and MLC phosphorylation (Birukov et al., 2003; Birukova et al., 2008a). However, changes in barrier integrity are not simply linked to changes in mechanical imbalance, as after cells have been re-plated to non-elastic substrates, they continue to be susceptible to leakage (Birukova et al., 2008b). Interestingly, pre-conditioning of ECs to CS provides protection against barrier breakdown (Birukova et al., 2008a), which is thought to be a result of induction of Rap1 signaling to promote junctional stability and suppress RhoA signaling (Ke et al., 2019). Interestingly, cyclic stretch can also induce EC proliferation in response to mechanical stimulation, in a process that requires cell–cell contacts. Indeed, stretch induced EC proliferation is lost upon VE-cadherin inhibition or knockdown (Liu et al., 2007; Neto et al., 2018) and dependent on the Hippo pathway downstream effector YAP (Neto et al., 2018).

Cardiovascular diseases such as hypertension and atherosclerosis are associated with CS, as an increase in blood pressure or changes in the ECM environment can affect the amount of stretch (Jufri et al., 2015). Pathological but not physiological CS results in activation of VEGFR2 and SFKs, and subsequent phosphorylation of VE-cadherin and disassembly of junctions (Gawlak et al., 2016; Tian et al., 2016a). Pathological and chronic exposure to CS also induces inflammation by upregulation of ICAM1 and secretion of IL-8 (Tian et al., 2016b). Therefore, increased CS can cause both pathological barrier breakdown and induce inflammation. Pulsatile stretch above 10% in brain ECs induces inflammation through ICAM1 and NO and induces amyloid precursor protein expression, which may explain why the severity of Alzheimer’s disease is increased with a higher pulsatility index and pressure (Nation et al., 2013; Gangoda et al., 2018). Interestingly, multi-directional stretch, analogous to the forces exerted on ECs in oscillatory FSS, results in upregulation of inflammatory stimulators such as NF-κB (Pedrigi et al., 2017), Both uniaxial and equiaxial stretch (10%) have been shown to drive induction of smooth muscle genes, an effect which is abrogated upon induction of FSS (uniform at 5 dyn/cm^2^ or cyclic between 0 and 5 dyn/cm^2^), suggesting stretch alone may induce transdifferentiation of ECs toward SMCs (Shoajei et al., 2014). Thus, multidirectional stretch may be directly linked to cellular processes which drive diseases such as fibrosis and atherosclerosis.

### Stretch in Lymphatic Endothelial Cells

Lymphatic vessels are exposed to stretch as a result of their fluid uptake and pumping properties. Capillaries take up interstitial fluid via an extrinsic (passive) mechanism, where the lymph enters the lymphatic capillaries against a hydrostatic pressure gradient (Scallan et al., 2016). It has been elegantly demonstrated that during early lymphatic development, fluid that leaks out of newly formed arteries swells the tissue that surrounds the first primary lymphatic structures and stretches these nascent structures (Planas-Paz et al., 2012). Stretching of the lymphatic ECs leads to activation of VEGFR3 and subsequent proliferation, resulting in enlargement of lymphatic structures so they are able to drain the excessive fluid from the embryonic tissue.

Collecting lymphatic vessels pump lymph via cyclic compression and expansion of lymphatic SMCs that surrounds the lymphatic collecting ECs (lymphatic muscle) (Zawieja, 2009). Increased intraluminal lymph pressure after fluid uptake leads to stretching of the collecting lymphatics and activates an intrinsic (active) lymph pump. The impact of stretch on lymphatic EC behavior exerted by SMC surrounding the collecting vessels has not been extensively addressed. In contrast to blood ECs, normal lymphatic ECs stretch is 4% elongation and pathological stretch is 8% elongation (Wang et al., 2017a), suggesting that similar to FSS, lymphatic ECs have a lower stretch setpoint.

To briefly summarize, blood and lymphatic vessels (and their vessel sub-types such as arteries, veins, capillaries and collectors) all display different sensitivities to mechanical stimuli, such as fluid shear stress, stiffness or stretch. These mechanical forces must be considered when performing *in vitro* experiments in the context of the vascular process to be studied, in order to gain biologically relevant insights into EC physiology.

## Advances in *In Vitro* Modeling of Vascular Forces

With an appreciation of the importance of the extracellular environment, the vascular field has begun to develop *in vitro* approaches that more closely mimic *in vivo* environments. Bioengineering approaches have been developed *in vitro* to recapitulate *in vivo* forces, providing more cost effective, biologically relevant experimentation. Here, we will review commonly used cell culture techniques to model forces of fluid shear stress, matrix stiffness and cell stretch, and discuss emerging technologies to combine these mechanical forces.

### 2D Techniques to Model FSS

**FIGURE 1:**
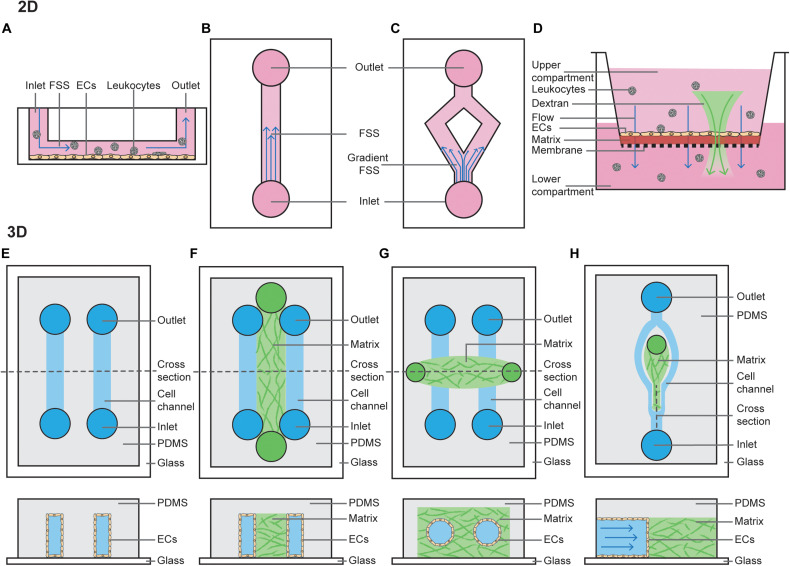
2D and 3D techniques to model FSS. **(A)** Cross section of a parallel plate flow chamber in which a monolayer of ECs is cultured on the bottom of the channel. Perfusion of media, containing growth factors and/or leukocytes, is added through the inlet and outlets. **(B)** Top view of a parallel plate flow chamber device to mimic steady laminar FSS or oscillatory laminar FSS, when fluid is pumped in a bidirectional manner. **(C)** Top view of a bifurcated channel to model gradient shear stress regions. **(D)** Cross section of a transwell filter system, where ECs are cultured on a thin ECM matrix on top of a porous membrane. The hydrostatic pressure difference between the upper and lower compartment models interstitial flow through the monolayer of ECs. Addition of fluorescent dextran or leukocytes to the upper compartment can be used to study permeability or leukocyte TEM. **(E)** Top view and horizontal cross section of a PDMS-only 3D microchannel in which ECs are grown inside the channels. **(F)** Top view and horizontal cross section of a PDMS-hydrogel hybrid 3D microchannel in which ECs are grown in the outer channels, and then allowed to migrate into the central hydrogel. **(G)** Top view and horizontal cross section of a hydrogel-only 3D microchannel in which ECs are grown fully surrounded by hydrogel. **(H)** Top view and vertical cross section of a PDMS-hydrogel hybrid 3D microchannel incorporating bifurcation.

There are numerous models which have been used in the vascular biology field to identify defined FSS-mediated EC outputs. Traditionally, these have been performed by seeding cells on a 2D flat and stiff substrate, allowing them to adhere and form a monolayer, then passing fluid over them (Figure 1) (Akbari et al., 2017). This can be achieved using a cone and plate system, where flow is generated by rotation of a cone around a central axis which is oriented in a perpendicular manner to the surface of a flat plate. More commonly, however, these experiments are performed using a parallel plate chamber, where fluid is pumped using a peristaltic pump, constant pressure head or syringe pump. These models have been particularly useful when modeling how ECs respond to changes in shear stress force and duration, where straight channels model laminar FSS (Figure 1B) and bifurcated channels model gradient shear stress regions (Figure 1C). Fluid can be pumped in a steady unidirectional manner or in an oscillatory manner, and in both of these cases it may be pulsatile. Examples of knowledge that has come from these models include when exposed to low levels of steady laminar FSS (10 dyn/cm^2^ for 5 h), EC junctions become stabilized (DePaola et al., 2001) and when exposed to disturbed shear stress, ECs acquire an inflammatory phenotype typically associated with atherosclerosis (Hahn and Schwartz, 2009; Chiu and Chien, 2011). Similar experiments have also helped to identify the mechanical contribution to formation of the lymphatic valves. When exposed to oscillatory but not steady state laminar FSS (4 dyn/cm^2^ or cultured on a rocking platform for 48 h), lymphatic ECs acquire features of lymphatic valves. In line with these findings, lymphatic valves form at sites of disturbed flow *in vivo* (Sabine et al., 2012, 2015; Kazenwadel et al., 2015; Cha et al., 2016).

Models of interstitial flow within lymphatic capillaries have been pioneered by the Swartz lab and are largely performed using Boyden chamber assays (Figure 1D) (i.e., transwell filter systems) or radial flow systems (Ng et al., 2004; Helm et al., 2005; Miteva et al., 2010; Swartz and Lund, 2012). Boyden chamber assays are performed by plating cells as a monolayer on a semi-porous ECM, such as collagen or fibrin, and seeded in the upper compartment of a transwell chamber. Then, a fixed hydrostatic pressure difference is maintained between the top and bottom chambers. Alternatively, radial flow chambers consist of ECM with ECs embedded and exposed to flow oriented radially, which is then placed between two glass coverslips and fixed with porous boundaries. Boyden chamber assays are also widely utilized amongst vascular biologists to study vascular permeability and chemotaxis of ECs and leukocytes (Nowak-Sliwinska et al., 2018).

2D FSS assays have been utilized for much of the pioneering work in understanding the mechanisms of how ECs sense flow-mediated mechanotransduction, and continue to provide valuable insights. However, these standard commercial assays are limited in that they do not fully recapitulate vessel physiology, which is significantly more complex. Of relevance to this review, these assays do not incorporate important components that can alter hydrodynamics (Huber et al., 2018) such as mural cells, ECM stiffness, or circular stretch.

### 3D Techniques to Model FSS

More complex microfluidic systems to model FSS involve growing ECs in 3D microchannels using microfabrication techniques. These can be entirely poly(dimethylsiloxane) (PDMS) derived, PDMS-hydrogel hybrids, or pure hydrogel (Akbari et al., 2017). Similar to 2D FSS models, flow can be controlled using hydrostatic pumps. PDMS-alone devices are typically linear rectangular channels that are optically transparent and permeable to gases, placed with an inlet-channel-outlet flow system (Figure 1E) (Duffy et al., 1998). These channels provide a suitable model to study dynamics of red blood cells or thrombosis (Shevkoplyas et al., 2003; Tsai et al., 2012). However, PDMS does not resemble native tissue environments as it is rigid, impermeable to water and not able to be remodeled by ECs (Stroock and Fischbach, 2010).

PDMS-hydrogel hybrids consist of a channel of hydrogel scaffold (typically collagen or fibrin) lined on both sides by PDMS channels lined with ECs (Figure 1F). These ECs then form sprouts and migrate across the hydrogel scaffolds in response to changes in pressure or growth factor gradients (Song and Munn, 2011), forming a perfused capillary plexus with a functional vascular barrier (Moya et al., 2013). ECs lining the PDMS channel can also be co-cultured with mural, stromal and cancer cells, and may be derived from either normal blood, tumor or lymphatic vessels (Kim et al., 2013, 2016). These hybrid models have provided extensive information about how capillaries sprout, revealing that angiogenesis and lymphangiogenesis occur in response to interstitial flow, as sprouts in this model preferentially extend against the direction of flow; i.e., capillaries sprout from vessels with a low pressure gradient toward those with higher pressure (Song and Munn, 2011; Song et al., 2012; Kim et al., 2016). Precisely how FSS applied to the PDMS channel mediates sprouting remains under debate. It has been shown steady laminar FSS (3 dyn/cm^2^ for 1–3 days) inhibits VEGF-induced sprouting (Song and Munn, 2011), whereas increasing the magnitude of FSS (to 10 dyn/cm^2^ for 24–48 h) can induce angiogenesis (Galie et al., 2014). This suggests a FSS setpoint may regulate the degree of sprouting.

Growing microvessels in pure hydrogels is perhaps the most physiological of all FSS models, as ECs form lumenised vessels surrounded by ECM. In this model, typically a PDMS mold is produced with two media ports at each end and an ECM port in the center (Figure 1G). The hydrogel scaffold is allowed to set within this 3D ECM scaffold around a cylindrical needle, before the needle is removed leaving a circular channel within the ECM (Polacheck et al., 2019). Cells (such as EC alone or EC co-culture with pericytes) can then be seeded via the media ports, where they will subsequently adhere and form a cylindrical monolayer (Polacheck et al., 2019). Once ECs have adhered, flow can be applied by a syringe pump or using a laboratory rocker (to produce steady laminar or oscillatory FSS, respectively). As a needle is used to set the channel, it’s width can be set to a defined diameter from 15–300 μm (Nguyen et al., 2013; Linville et al., 2016; Polacheck et al., 2017). This is unlike vessels in PDMS-hydrogel hybrids, where form lumens spontaneously. These systems can be used to assess barrier function of the vasculature. Interestingly, in the absence of flow these vessels are leaky but when constant application of low FSS is applied (5 dyn/cm^2^ overnight), cell–cell adhesion is induced to prevent leakage of 70-kDa Dextran (Polacheck et al., 2017). If two cylindrical vessels are embedded within the same hydrogel these models can also be used to assess angiogenesis, with newly formed capillaries sprouting across the ECM in response to growth factor gradients between two larger vessels (Nguyen et al., 2013), a process which is dependent on EC-mediated Myosin IIA forces (Yoon et al., 2019).

While hydrogel-embedded microvessels are excellent models to study the effects of FSS on the vasculature, cells are embedded in a linear vessel. Therefore, they do not accurately model the effects of disturbed flow in bifurcating vessels which are found abundantly *in vivo*. Recently, PDMS-hydrogel hybrids have been developed to study the effects of flow at bifurcation points in vessels (Figure 1H) (Akbari et al., 2018). Interestingly both angiogenic sprouting and permeability at bifurcation points is reduced after 48 h (where the bifurcation point has stagnation pressure of approximately 38 dyn/cm^2^ and branched vessels are exposed to 3 dyn/cm^2^) (Akbari et al., 2018, 2019). This suggests bifurcation points stabilize the blood vasculature, as has been demonstrated *in vivo* (Ghaffari et al., 2015).

### Models to Study EC Behavior Regulated via Matrix Stiffness and Stretching

**FIGURE 2:**
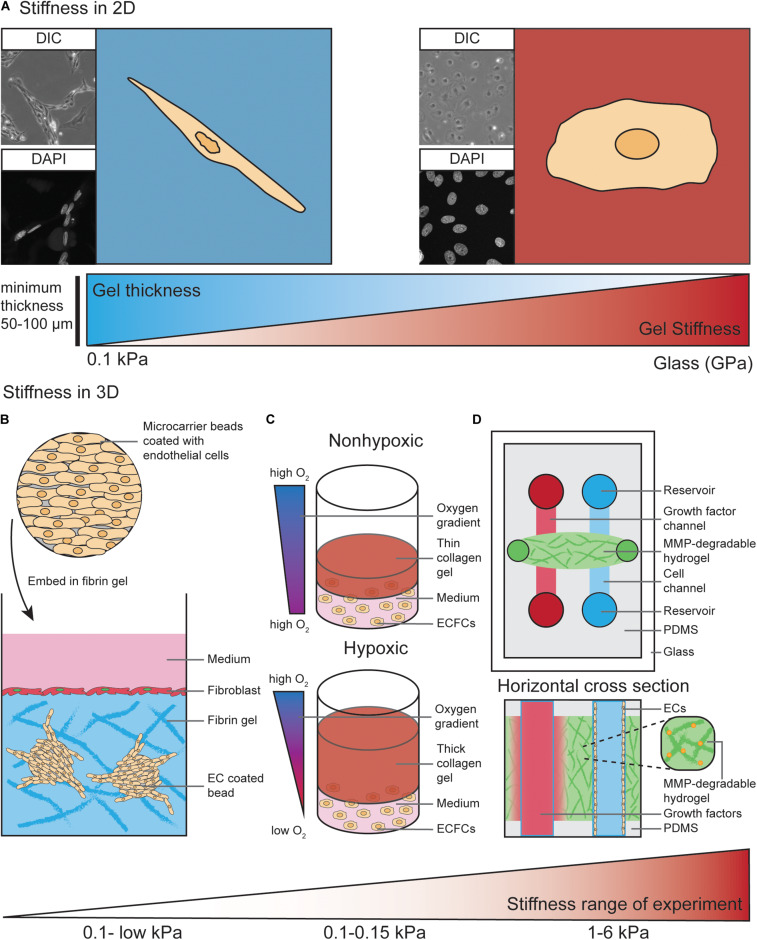
Models to study EC behavior regulated by matrix stiffness. **(A)** ECs cultured on 2D substrates with different stiffness ranging from 0.1 kPa up to GPa (glass) display different morphologies. As an example, on soft substrates human dermal lymphatic endothelial cells (HDLECs, C-12216, PromoCell, Heidelberg, Germany) show a spindle-shaped morphology with a distorted and elongated nucleus and form networks via their cell–cell interactions. On stiff substrates HDLECs flatten out and preferably undergo cell-substrate interactions compared to HDLECs cultured on soft substrates. ECs can “feel” up to several micrometers deep into a compliant substrate, suggesting a minimum hydrogel thickness of 50–100 μm is necessary to analyze ECs on compliant 2D substrates. **(B)** ECs coated on cytodex microcarrier beads are embedded into a fibrin hydrogel to study 3D *in vitro* angiogenic sprouting. Fibroblast are layered on top of the gel to provide necessary factors to promote EC sprouting and lumenisation. The stiffness range of this assay is very low due to the use of natural materials such as fibrin or collagen. **(C)** Encapsulation of endothelial colony-forming cells (ECFCs) in 3D collagen gels with varying thickness provides a temporal hypoxic gradient, where thick gels model hypoxia and thin gels normoxia. Increased matrix stiffness of the 3D collagen gels is performed by crosslinking and results in a stiffness range of 0.1–0.15 kPa. **(D)** Top view and horizontal cross section of PDMS-hydrogel microchannel containing non-swelling MMP-degradable DexMA hydrogel. This allows ECs grown in one channel to migrate toward a growth factor gradient formed by diffusion of growth factors from the other channel. Incorporation of the MMP-degradable peptide crosslinkers into the synthetic hydrogel makes the stiffness variable within a range of 1–6 kPa, compared to non-variable standard hydrogels used in Figures 1F–H.

Conventional *in vitro* studies have long been performed using plastic or glass matrices to culture ECs. The stiffness of plastic and glass dishes (which is in the GPa range) by far exceeds the physiological stiffness that ECs experience *in vivo* (Wells, 2008; Guimarães et al., 2020). As discussed above, the physiological ECM stiffness experienced by ECs varies from very soft microenvironments during development to stiffer microenvironments in arterial ECs or in aged and diseased tissues. EC behavior in response to more compliant substrates has been assessed more frequently in 2D culture where ECs are seeded on top of those substrates (Figure 2A).

Hydrogels, which are water-swollen networks of polymers, have emerged as the most promising choice for cell culture since they mimic salient elements of the ECM, have mechanics similar to those of softer tissues and can support cell adhesion and protein sequestration (Tibbitt and Anseth, 2009). The properties of hydrogels, such as mechanics, swelling, porosity and degradation, are modifiable and can be adjusted to the specific requirements of the desired experiment. For commercially available hydrogels, these properties have been experimentally characterized and can be easily adapted by the user (Caliari and Burdick, 2016). Diverse stiffness hydrogels can be generated from natural materials, such as collagen, fibrin or alginate, and synthetic materials, such as polyacrylamide (PA) or Polyethylene glycol (PEG) (Caliari and Burdick, 2016). In selected settings, the use of natural materials can be advantageous as they exhibit high biocompatibility, allowing for EC interaction via integrin ligation. However, ECM stiffness provided by those natural materials is very soft. For example, the matrix stiffness of collagen hydrogels is very low (<1 kPa) (Mason et al., 2013). Another frequently used natural hydrogel, Matrigel, which is a heterogenic mixture of various ECM proteins, was found to have a stiffness of approximately 0.45 kPa when measured with AFM in an aqueous, temperature controlled environment (Soofi et al., 2009). Furthermore, Matrigel and other natural hydrogel materials do not allow user-defined variability of ECM components.

In contrast, synthetic materials are more inert and usually have to be combined with chemically modified crosslinkers. This allows for integrin ligation to facilitate interaction of ECs with the substrate (Caliari and Burdick, 2016). Synthetic materials, such as PA, can be utilized to generate hydrogels with much wider matrix stiffness ranges, from 0.1 kPa to high kPa values (Tse and Engler, 2010; Denisin and Pruitt, 2016). These characteristics become highly advantageous when matrix stiffness and EC-matrix interactions have to be analyzed separately. For example, PA hydrogels with different matrix stiffness values can be generated with variable collagen I concentrations. This allows for study of the balance between substrate mechanics and matrix chemistry in the process of EC network assembly (Califano and Reinhart-King, 2008).

The impact of ECM stiffness on EC behavior has not been studied extensively in 3D *in vitro* models. Most 3D studies to analyze angiogenic sprouting processes have been performed using natural hydrogel materials, such as collagen type I and fibrin (Juliar et al., 2018). For example, ECs can be coated onto cytodex microcarriers and embedded into a fibrin hydrogel (Figure 2B). Here, fibroblasts are layered on top of the gel where they provide necessary soluble factors that promote EC sprouting from the surface of the beads (Nakatsu et al., 2007). These fibroblast derived factors act to minimally stiffen gels (0.03–0.05 kPa) and are required for the lumenisation of sprouting vessels (Newman et al., 2011). In an elegant study by Blatchley et al. (2019), thick 3D collagen gels were demonstrated to provide a temporal hypoxic gradient (Figure 2C), allowing for the study of EC behavior in response to changes in both hypoxia and ECM stiffness. These models have simultaneously revealed encapsulation of endothelial colony-forming cells (ECFCs) leads to vasculogenic cluster formation through ROS production in hypoxic (thick) but not normoxic (thin) collagen hydrogels (Figure 2C), and that increased matrix stiffness via collagen crosslinking leads to reduced cluster formation (Blatchley et al., 2019).

As described above, natural hydrogels provide a soft microenvironment for ECs in culture, suggesting these 3D *in vitro* models are most suitable to mimic the soft ECM environment that allows ECs to migrate and sprout *in vivo* (Mammoto et al., 2009; Frye et al., 2018). To generate stiffer (>1 kPa) 3D environments to study the impact of higher ECM stiffness on EC behavior, commonly used PA hydrogels are not suitable to encapsulate ECs due to the high toxicity of the hydrogel precursors (Caliari and Burdick, 2016). Therefore, synthetic PEG hydrogels are more widely used to analyze the effects of a stiff ECM in a 3D context (Jonker et al., 2015; Carthew et al., 2018; Whitehead et al., 2018).

In addition to higher matrix stiffness ranges and the ability to independently modulate adhesive ligand presentation, tunable degradation of synthetic hydrogels offers the possibility to alter the microenvironment and stiffness experienced by cells during an experiment. This can be done using ultraviolet light (Kloxin et al., 2009) or by adding engineered, MMP-degradable peptide crosslinkers to the hydrogel solution (Lutolf et al., 2003). Another advantage of using synthetic materials is that the swelling properties of these hydrogels can be modulated. An elegant study by Trappmann et al. (2017) eliminated the swelling properties of a synthetic hydrogel after polymerization, which enabled its integration into microfluidic channels (without channel closure) and subsequent channel colonization with ECs (Figure 2D) (Trappmann et al., 2017).

In addition to alterations in stiffness, growing cells on flexible substrates to allow for cyclic stretch has wide implications for EC biology. Most of the studies on stretch are performed using commercial systems, such as the Flexcell Tension Plus system (Flexercell, United States). Here, cells are plated on flexible silicon membranes coated with matrix and grown in standard media before being subjected to stretch. These systems induce cellular elongation in response to stretching and deformation of the elastomere base of the plates, achieved using vacuum pressure which exposes cells to radial, but not uniaxial stretch (Gilbert et al., 1994). High throughput techniques to induce CS in a 96 well place format have been developed using PDMS devices, where cells are exposed to uniaxial stretch. However, these can only reach 10% stretch (Matsui et al., 2018). Advances in models for measuring cyclic stretch are found in lung-on-a-chip models, discussed in further detail in the following section.

## Integrative and Novel Approaches to Model and Visualize EC Forces

### FRET Biosensors to Visualize Tensile Changes

**FIGURE 3:**
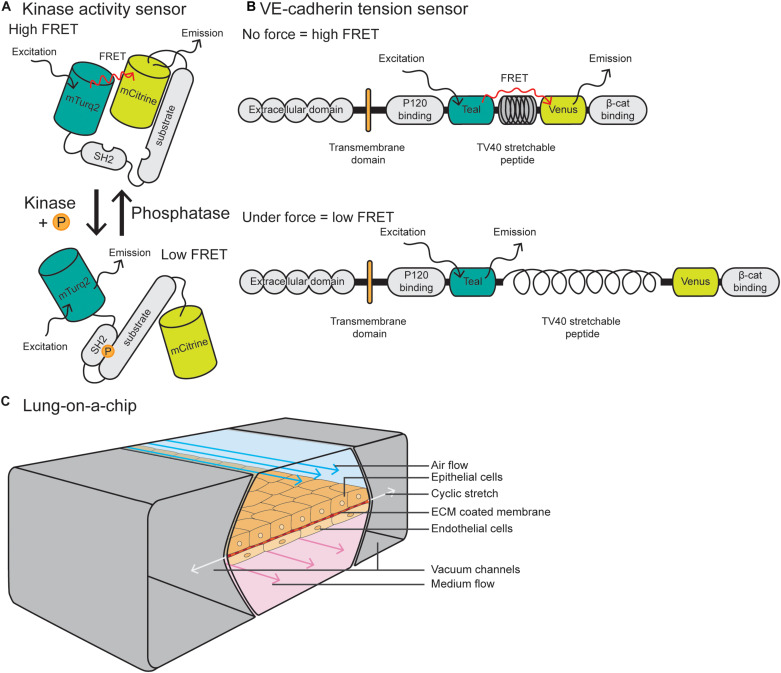
Integrative and novel approaches to study EC forces. **(A)** Schematic of a Src kinase activity FRET biosensor containing mTurqoise2 as donor and mCitrine as acceptor. Upon kinase activation, the substrate becomes phosphorylated and induces intramolecular binding of the SH2 domain, resulting in a conformational change of the molecule and reduction of FRET efficiency. **(B)** The VE-cadherin FRET tension sensor consists of the VE-cadherin protein and the sensor module, containing Teal as donor and Venus as acceptor, connected by the TV40 stretchable peptide. The FRET sensor module was placed in between the p120-catenin and β-catenin binding sites. When there is no force applied on VE-cadherin, there is a high FRET efficiency, while force on the p120-catenin and β-catenin binding sites stretches the TV40 linker and spatially separates the Venus acceptor and Teal donor, resulting in lower FRET efficiency. **(C)** Schematic of a lung-on-a-chip model in which alveolar epithelial cells are plated on the apical side of an ECM-coated porous membrane, and EC are plated on the basal side. The epithelial cells are exposed to air flow and ECs are exposed to fluid flow. Cyclic stretch is applied by a vacuum in the two flanking chambers, resulting in extension and retraction of the epithelial/endothelial cell layer in the center.

Förster resonance energy transfer (FRET) reporter constructs have been widely used in the field of cell biology to study protein activity, interactions and conformational changes. FRET biosensors rely on transferring energy from the excited state of the donor fluorescent protein to the unexcited acceptor fluorescent protein, which then emits free photons (Snell et al., 2018). FRET efficiency is sensitive to the distance and orientation of the separation of the two fluorophores which make up the FRET pair, with distance changes as small as 1 nm substantially affecting the amount of energy transfer. Biosensors may consist of an intracellular construct that is sensitive to kinase activity, such as Src and FAK-Lyn (Figure 3A) (Wang et al., 2005; Seong et al., 2011) or conformational changes in proteins, such as Src, non-muscle Myosin II and α-catenin (Kim et al., 2015; Markwardt et al., 2018; Koudelkova et al., 2019). Biosensors have also been utilized *in vivo* to measure changes in protein activity, such as Src, AKT, NO, and RhoA (Nobis et al., 2014; Li et al., 2017; Nobis et al., 2017; Conway et al., 2018; Bhuckory et al., 2019).

The vascular biology field has utilized FRET biosensors in EC culture to measure tension across vinculin and talin at FAs (Grashoff et al., 2010; Kumar et al., 2016) and PECAM-1 and VE-cadherin at cell–cell junctions (Conway et al., 2013). These tension sensor constructs consist of a donor fluorophore connected to an acceptor through a nanospring derived from the elastic spider silk protein flagelliform (Figure 3B) (Grashoff et al., 2010). Where tension is low or absent, the nanospring is compact and FRET is high, and conversely, application of tension stretches the spring and decreases FRET. These tension sensors are now being utilized *in vivo* in ECs, with a VE-cadherin-tension sensor zebrafish recently described by Lagendijk et al. (2017). This zebrafish line allows for live, intravital fluorescence lifetime imaging microscopy–fluorescence resonance energy transfer (FLIM- FRET) imaging of tension across junctions. These models and imaging techniques are predicted to be more widely utilized in the vascular biology field to measure changes in tension or subcellular signaling dynamics in tissue culture or *in vivo* models.

### Organ-on-a-Chip

Recent scRNA-seq approaches have revealed high levels of EC heterogeneity not only between EC types, but also between organ tissues (He et al., 2018; Vanlandewijck and Betsholtz, 2018; Takeda et al., 2019; Kalucka et al., 2020). To investigate organ-specific vasculature functions, organ-on-a-chip models have been developed to study tumor, gut, liver, pancreas and muscle function. These are also being applied in the field of personalized medicine by incorporating patient samples (van den Berg et al., 2019). These models aim to culture cells in conditions that closely mimic the body, taking into account factors such as tissue geometry and composition, dynamics and flow gradients (Bhatia and Ingber, 2014). Generally, they consist of microfluidic devices integrated with microchip manufacturing methods, which allow for continuously perfused chambers with live cells that have been arranged to simulate tissue- and organ-level physiology.

Due to the fact that the lung vasculature is exposed to multiple mechanical forces, here we will focus on lung-on-a-chip models. Pioneered by the Ingber laboratory, these devices are comprised of alveolar epithelial cells plated on the apical side of an ECM-coated porous membrane, with EC coated on the basal side (Huh et al., 2010; Huh et al., 2013) (Figure 3C). Alveolar cells are exposed to air and ECs are exposed to FSS at a range between 1 and 15 dyn/cm^2^, similar to forces applied *in vivo*. To model cyclic stretch, suction is applied to two external chambers flanking the cell channel, causing it to extend and retract at a rate and degree to that found in the body (approximately 10% cyclic stretch). This allows for modeling of multiple mechanical forces on the cells, including stretch, ECM components and fluid flow, making them superior to traditional microfluidic devices. Such devices can also can be modified to model other tissues with polarized EC interfaces, such as kidney (EC-podocyte) (Musah et al., 2017), liver (EC-hepatocyte) (Jang et al., 2019), brain (EC-astrocyte) (Herland et al., 2016), and tumor (tumor-epithelium-EC) (Hassell et al., 2017).

Microcirculation-on-a-chip models have been developed to model organizational layers within the vasculature. One such model allows for the co-culture of blood and lymphatic vessels within the same device, separated by a porous polyethylene terephthalate (PET) membrane (Sato et al., 2015). This chip can be utilized to measure vascular permeability and lymphatic absorption. Other devices with an organized composition of ECs and SMCs separated by a porous membrane have been developed to better model the composition of the arterial wall. These allow for a more accurate examination of the interactions and signaling between these cellular components (van Engeland et al., 2018). Finally, models of interconnected, perfused vessels with a high flow arterial input, capillary plexus and low flow venous output have also been developed, to model multiple stages of vascular development including vasculogenesis, angiogenesis to anastomosis (Wang et al., 2016). While advantageous for their complex vascular organization, these chips lack the integration of other organ-specific cells.

In addition to organ-on-a-chip models, the use of organoids for *in vitro* investigation of organ-specific phenotypes is emerging in the fields of biology and drug discovery. A major road block in organoid research is how to incorporate a functional vasculature network. Not only is the field faced with the challenge of EC heterogeneity and matching the vasculature to the type of tissue organoid, organoids *in vitro* remain small and immature due to our inability to grow a perfused, functional vascular network within them. However, when transplanted *in vivo*, organoids can become vascularized due to ECs derived from transplanted iPSCs or the host (Pham et al., 2018; van den Berg et al., 2018). For a detailed review on the challenges in vascularization of organoids, see the review by Daniel and Cleaver (2019).

## Summary

The complexity and relevance of mechanical forces to generate and maintain a functional vascular system is well appreciated by vascular biologists. Sophisticated methods have been developed to model and measure forces in 2D cultured monolayers, but are now being adopted into more complex 3D models. The choice of a suitable 2D or 3D model is dependent on the specific endothelial process to be studied. While 3D models may be superior to study FSS within a model of a circular, lumenised vessel, or to model the ECM stiffness microenvironment surrounding sprouting and migrating ECs, stiffness experienced by ECs of a mature vessel network may be more accurately studied in 2D settings. This is due to the fact mature vessels are equipped with a monolayer of ECs which are naturally attached to the underlying ECM in a 2D manner.

Given the transport of fluid exposes ECs to the mechanical forces of FSS and cyclic stretch simultaneously, and the stiffness of the surrounding matrix will affect the degree of cyclic stretch and FSS, techniques to incorporate modeling of all these forces will yield maximal physiological relevance. We believe that further improvements to *in vitro* and *in vivo* force modeling will begin to unravel the precise impact of FSS, stiffness and stretch on the development and function of a heterogeneous vessel network across different tissues.

## Author Contributions

MF, EG, and LS wrote and edited the manuscript.

## Conflict of Interest

The authors declare that the research was conducted in the absence of any commercial or financial relationships that could be construed as a potential conflict of interest. The handling editor declared a past co-authorship with one of the authors LS.
